# On Some Affections of the Thyroid

**Published:** 1891-10-10

**Authors:** 


### SURGERY.

### ON SOME AFFECTIONS OF THE THYROID.

The thyroid gland is subject to enlargements, some of which are analogous to those occurring in other glandular bodies, while some appear to be peculiar to it alone. As a highly vascular organ it may show temporary or permanent increase of size from increased blood supply, and from dilatation of the vessels, whether this be caused by a vasomotor paralysis, or by an impediment to the return of blood. The hypercœmia may lead to hypertrophy, to increase of the cells in size and number, or to increase of the colloid substance contained in those cells.

The hypertrophy, however, may arise without, so far as we know, any hypercœmia as an independent process, though an increased vascular supply may be formed subsequent to the enlargement. The gland again is subject to the invasion of various neoplasms, such as malignant growths, much as the rest of the body is. In other glands the effect of hypertrophy is quickly shown by interference with function, but since we are very much in the dark as to what the true functions of the thyroid are, it is difficult to discover the presence of minor forms of hypertrophy and their effects other than the mechanical ones produced in neighbouring organs. Of late years much attention has been been given to the diseased states following removal or absence of the gland, viz., cachexia strumipriva, and myxœdema; and a considerable number of facts have been ascertained which throw light on the functions of the normal gland. If we put on one side these diseases, together with the affections characterized by enlargement, exopthalmic goitre, and those connected with ordinary malignant growths, we find very few pathological states of the thyroid which call for our consideration. There are, perhaps, no conditions similar to many kinds of degeneration which affect the kidney and liver. Rare cases of acute thyroiditis or of syphilitic and tubercular disease are nearly all the failings which have usually been ascribed to this organ. The study of myxœdema, however, tends to show that a numerous body of affections, generally ascribed to the vascular and nervous systems, are connected with thyroid troubles, for the milder forms of myxœdema are much more common than wa imagined some years ago, and their cause is rarely suspected in the absence of a minute examination of the thyroid.

The ordinary enlargements and their treatment have been recently discussed by Mr. Berry, who has devoted minute research to every aspect of the question. The causation of ordinary goitre has long been a moot question. The endemic character in a large proportion of [the cases points clearly to some external local cause. A high or low temperature, dampness, altitude, want of light and air appear to have nothing to do with it. Mr. Berry has shown that the disease in England chiefly occurs on the carboniferous limestones, and to some extent on other calcareous rocks and sandstones. The magnesian limestone does not, however, appear specially connected with it, nor is the mere hardness of the water apparently a cause by itself. Yet there is, on the other hand, much evidence that the poison is often conveyed in the drinking water. "Such water is usually, if not always derived from calcareous soils; but it is probable that the goitre-producing poison is not a salt of lime or manganese." There are, indeed, springs known the water of which cause goitre in a few weeks' time when drunk. Researches have been made to discover whether such water contains any micro-organism capable of producing the disease, but the results have so far been untrustworthy. Mr. Berry inclines to the belief that the efficient agent is a mineral one, neither a salt of lime or iron, but of one of the alkalies or alkaline earths. Bircher and Klebs hold the view that it is due to an organic substance which develops like malaria only in certain localities, and which is conveyed by drinking water either as a ptomaine or some other alkaloid.

Occasionally goitre appears to become epidemic, and attacks large numbers of persons in a very short time. This would rather be an argument in favour of its organic origin; yet, so far as we know, there is no evidence that it is ever communicated from one person to another or from parent to child. The malignant and innocent forms are so closely allied, and so often mixed in the same person, that they are probably due to the same exciting cause, which attains different degrees of intensity.

The treatment of parenchymatous and fibroid goitre may include removal of the individual from the goitrous district (if it be an endemic case), blistering, or the administration internally of fluoric acid. Great success has been claimed for the application of binodide of mercury ointment, but the results in this country have hardly borne out the expectations. In parenchymatous and cystic goitres, the injection of iodine and of iron solutions has found many advocates. Mr. Berry is in favour of Semon's method of iodine injections, but he shows that fatal results are not uncommon, even where every care is taken. Von Moorhof has recently recommended injections of iodoform dissolved in ether and oil. They appear to be less dangerous than the ordinary iodine ones, and in seventy-nine cases where he used them they were followed by some reduction in size of the tumour. Treatment by setons is nearly given up, from the risk of wounding veins and from the danger of pyæmia.

Ligature of the thyroid arteries has been revived of late, and both the superior and inferior vessels have been tied at once; but the latter are difficult to reach, so that the operation is a serious one. The cases most suitable for it are those of parenchymatous enlargement in the early stage. The thyroid isthmus has been divided for the relief of dyspnœa. Sometimes it succeeds by allowing the separation of the two lobes, and at other times by permitting the escape of the colloid secretion; but Mr. Berry points out that it is insufficient to relieve the cases of most urgent dyspnœa, and often the goitre reappears when the wound has healed.

The most important method after all of treating parenchymatous goitres is extirpation. The removal of the whole gland is now almost given up on account of the danger of cachexia strumipriva, and against resection of the isthmus there are the same objections as against division of it. Partial extirpation has been carried to great perfection, and with very satisfactory results.

For cystic goitres we may employ tapping, with or without drainage or injection. Of late the method of enucleation has been applied to them, and to those solid tumours which have a capsule, and which are either single or only few in number. The advantages are less danger of cachexia, and less risk of wounding the nerves or great vessels. However, there is greater likelihood of recurrence than when part of the gland is excised. Where the tumour is single, embedded in healthy tissues and easily shelled out, enucleation may be preferred to the more radical operation.

With regard to the risk of cachexia after complete extirpation, it has been shown that about one in four became affected out of 277 cases, but the probability is that the gland was not completely extirpated in a large number of the cases. If so, the risk in really complete removal is much higher. In partial thyroidectomy the same risk occurs in a less degree. Only six cases out of 550 were noted by the committee of the Clinical Society, and, probably, either the portion left behind was very small or was thoroughly diseased. As a rule, a healthy portion of gland left behind increases in size, and appears to do the work of the entire organ.

